# Exercise improves body composition, physical fitness, and blood levels of C-peptide and IGF-1 in 11- to 12-year-old boys with obesity

**DOI:** 10.3389/fphys.2024.1451427

**Published:** 2025-01-02

**Authors:** Min-Seong Ha, Hyo Youl Moon, Minchul Lee, Jang Soo Yook

**Affiliations:** ^1^ Laboratory of Sports Conditioning: Nutrition Biochemistry and Neuroscience, Department of Sport Science, College of Arts and Sports, University of Seoul, Seoul, Republic of Korea; ^2^ Department of Physical Education, Seoul National University, Seoul, Republic of Korea; ^3^ Department of Sports Medicine, College of Health Science, CHA University, Pocheon, Republic of Korea; ^4^ Institute of Sports and Arts Convergence, Inha University, Incheon, Republic of Korea

**Keywords:** exercise, childhood obesity, physical fitness, C-peptide, IGF-1

## Abstract

**Introduction:**

Exercise is vital in preventing and treating obesity. Despite its importance, the understanding of how exercise influences childhood obesity at the biochemical level is limited. In this study, we explore the effects of a 16-week exercise program (EP) on body composition, physical fitness, and the blood levels of hormones related to obesity.

**Methods:**

Sixteen boys with obesity (n = 16) and seventeen boys without obesity (n = 17) took part in an EP comprising sports games and aerobic and resistance exercises. We examined alterations in body composition and physical fitness. In addition, we measured circulating hormone levels, including C-peptide, resistin, insulin-like growth factor 1 (IGF-1), and growth hormone (GH), in the blood.

**Results:**

Body fat percentage (BFP) decreased from 37.61% at pre-EP to 29.16% at post-EP in the obese group, but not in the non-obese group. The EP decreased C-peptide (4.58 ng/mL vs. 2.96 ng/mL, *p* < 0.001) and resistin levels (14.05 ng/mL vs. 11.06 ng/mL, *p* < 0.001) in the obese group. After the EP, significant improvement in IGF-1 (non-obese: 265.56 ng/mL vs. 311.81 ng/mL, *p* < 0.001; obese: 224.74 ng/mL vs. 272.89 ng/mL, *p* < 0.001) and GH levels (non-obese: 3.91 ng/mL vs. 4.80 ng/mL, *p* < 0.05; obese: 1.76 ng/mL vs. 2.51 ng/mL, *p* < 0.05) were observed in both groups. Lower C-peptide levels were associated with BFP (*r* = 0.447, *p* = 0.009) and muscle mass (*r* = −0.385, *p* = 0.02), whereas enhanced IGF-1 levels correlated with increased muscle strength (*r* = 0.343, *p* = 0.05) and cardiovascular fitness (*r* = 0.347, *p* = 0.04). Multiple linear regression analysis revealed that cardiovascular fitness variability and BFP in the obese group were determined by C-peptide (β = −0.054, *p* < 0.001) and IGF-1 levels (β = −2.936, *p* < 0.05), respectively.

**Discussion:**

Exercise may induce positive effects on improvements in body composition and physical fitness, as well as on blood levels of metabolic biochemicals such as C-peptide and IGF-1, in adolescent boys with obesity.

## 1 Introduction

Childhood and adolescence overweight or obesity represent significant global health concerns, constituting a global public health challenge amid a shared obesogenic lifestyle and contributing to both physiological and psychological challenges throughout an individual’s life (Lobstein et al., 2015; Cesare et al., 2019). The prevalence of childhood overweight and obesity has surged worldwide in recent years (Abarca-Gómez et al., 2017). As of 2016, a World Health Organization (WHO) report indicated that over 340 million children and adolescents, aged 5–19 years, were estimated to be overweight or obese. Moreover, childhood obesity is strongly linked to an elevated risk of mortality and the premature onset of chronic diseases, including type-2 diabetes, in adulthood (Abdullah et al., 2011; Sabin et al., 2015). Consequently, addressing excess weight during childhood and adolescence through lifestyle modifications is imperative for prevention and reversal (Salam et al., 2020).

Although the etiology of childhood obesity is multifaceted, it predominantly results from overconsumption and decreased energy expenditure (Woodruff et al., 2009). A high level of physical activity has been suggested as a significant lifestyle modification to positively affect the energy balance equation (Westerterp, 2017; Wyszyńska et al., 2020). In addition, once obesity takes root in childhood, it tends to escalate by increasing the size and number of adipocytes; making overall body fat mass challenging to manage through exercise or diet alone (Raine et al., 2017; Zamora-Mendoza et al., 2018). Longitudinal studies have shown that adolescents who were physically active are less likely to become overweight in adulthood (Menschik et al., 2008). Notably, recent studies have indicated a further surge in childhood obesity rates since the onset of the 2019 coronavirus pandemic, owing to school closures and lockdowns that limited opportunities for physical activity (Jenssen et al., 2021). To address this global epidemic, the WHO has advocated for non-pharmacological interventions to mitigate physical inactivity (Waxman and Assembly, 2004). Furthermore, global guidelines published by the WHO suggest that children should engage in at least 60 min of moderate-to-vigorous physical activity daily (Chaput et al., 2020). Therefore, exercise intervention with increasing physical activity levels has been identified as a preventive and therapeutic measure for childhood overweight and obesity.

Numerous studies focusing on children and adolescents with overweight and obesity consistently highlight the positive impact of regular exercise on body composition, including body mass index (BMI) score, as well as improvements in metabolic abnormalities and biochemical variables, such as insulin resistance markers, inflammatory markers, and adipocytokines (Alberga et al., 2015; Kelley et al., 2015). Furthermore, our recent previous study identified that body composition is significantly correlated with physical fitness levels, including strength and agility, according to the degree of obesity classified based on BMI in children. This correlation further impacted the effects of exercise intervention depending on the degree of obesity (Lee et al., 2022). Consequently, expert consensus recommendations emphasize the importance of an appropriate exercise program (EP) for the effective prevention and improvement of obesity in children and adolescents.

Childhood obesity is typically categorized by an age-specific BMI for an appropriate reference population (Cole et al., 2000). However, relying solely on this anthropometric diagnosis, which is a rough measurement, may cause lack of precision in identifying individuals at a heightened risk of disease development. Consequently, an increasing body of literature proposes an alternative approach using obesity biomarkers, such as circulating hormones (e.g., adipokines, insulin, and insulin-like growth factor [IGF-1]), to delineate obesity phenotypes associated with an elevated risk of morbidity and mortality. These biomarkers also serve as valuable tools to monitor and implement intervention programs including physical activity (Nimptsch et al., 2018).

Clinical studies and experimental data indicate that C-peptide, a biological peptide (Wahren et al., 2007), has been traditionally viewed as an indicator of the pancreatic secretion ability or an indirect measure of β-cell function, making it a focal point in diabetes-related investigations (Polonsky et al., 1986). Furthermore, C-peptide exhibit physiological activity, functioning as peptides that act independently of insulin (Garcia-Serrano et al., 2015). In studies involving skeletal muscle, both insulin and C-peptide demonstrated equal effectiveness in mediating glucose uptake through insulin signaling pathways (Zierath et al., 1991). Moreover, C-peptide bind to the cellular membrane of adipocytes, influencing changes in adipocytokine secretion, thereby potentially playing a role in regulating adipose tissue within the context of human energy homeostasis (Garcia-Serrano et al., 2015). In addition, they contribute to the regulation of body composition and inflammatory biomarkers (Bo et al., 2005; Gishti et al., 2015). As a result, C-peptide has emerged as a potentially relevant biomarker of obesity.

In addition to exercise demonstrating positive effects on anthropometric indices of obesity progression, such as BMI (Kelley et al., 2015), its advantages extend to reducing insulin resistance and β-cell dysfunction, as evidenced by improvements in the disposition index among adolescents with overweight and obesity (Shih and Kwok, 2018). Furthermore, a longitudinal study has indicated a significant correlation between high physical activity and decreased β-cell function (assessed through serum C-peptide levels) in lean children (Huus et al., 2016). This suggests that exercise may regulate obesity-related biomarker hormones to mitigate childhood obesity.

A systematic review and meta-analysis revealed that the most effective approach to preventing childhood obesity in children aged 6–12 years is school-based interventions that incorporate both aerobic and resistance exercises (Podnar et al., 2021). Moreover, several previous studies have shown that combined resistance and aerobic exercise (CRAE) interventions, lasting 8–18 weeks, have a positive impact on body composition, physical fitness, and blood levels of metabolic hormones (leptin, adiponectin, and insulin) (Jeon et al., 2013; Lopes et al., 2016; Bharath et al., 2018). However, whether the improvement in body composition and physical fitness in children with obesity achieved by CRAE is regulated by such metabolic factors in the blood remains unclear. Therefore, the present study examined the effects of a 16-week EP, including CRAE, on body composition, physical fitness levels, and circulating hormones, including C-peptide, resistin, IGF-1, and growth hormone (GH), in adolescent boys with obesity. Additionally, to investigate a potential contributor of obesity-related circulating biomarker hormones response to the EP, we assessed serum levels of these hormones and their changes in body composition and physical fitness levels after the EP intervention using correlation coefficients and stepwise multiple linear regression analysis.

## 2 Materials and method

### 2.1 Participants and study design

The ideal sample size was calculated using G-power software (version 3.1; Kiel University, Kiel, Germany), with parameters set at an effect size of 0.25 (default), a significance level (alpha) of 0.05, and a power (1-beta) of 0.80. This computation indicated that 34 participants were needed. However, to account for potential dropouts, 42 participants were initially recruited. Owing to various reasons, nine participants withdrew, resulting in a final sample size of 33 individuals. A total of 33 elementary school students, aged 11–12 years (mean age 11.42 ± 1.12 years; all boys), actively participated in this study. Participants were stratified based on BMI scores with age- and sex-appropriate cutoff values, resulting in two groups: non-obese (BMI: 18–24; n = 17) and overweight and obese groups (BMI: 25–32; n = 16) (Onis et al., 2007). Comprehensive health assessments were conducted through a health questionnaire, physical examination, and laboratory tests. Before participation, parents of the children willing to participate were given a detailed explanation of the objectives and intentions of the study, and all provided written informed consent. The exclusion criteria were having received drug therapy in the past 6 months, having engaged in regular exercise, or having had previous musculoskeletal disease. During the study period, the use of medications that could affect the experiment was restricted. Physical fitness test, body composition parameters, and blood samples were measured using the same methods under identical conditions. For the measurements, participants were required to maintain a minimum of 8 h of fasting, with no vitamin intake or vigorous exercise. The assessments were conducted twice for both the exercise and control groups (during the pre-16-week and post-16-week rest periods), following the procedures recommended by the American College of Sports Medicine (Thompson et al., 2013).

### 2.2 Exercise program

The EP was adapted into a structured and supervised after-school EP based on the exercise prescription program outlined by the American College of Sports Medicine (Thompson et al., 2013; Lee et al., 2022). The program was conducted three times per week during a 2-week adjustment period. Each session lasted 60 min, including a 5-min warm-up, a 50-min main exercise period (comprising sports games and aerobic and resistance exercises), and a 5-min cool-down. The intensity was gradually increased: from 50% to 60% of heart rate reserve (HRR)/11–12 on the rated perceived exertion (RPE) scale in weeks 1–4, to 60%–70% of HRR/12–13 of RPE in weeks 5–13, and finally to 70%–80% of HRR/13–14 of RPE in weeks 14–16. To ensure proper training intensity, each participant wore a heart rate monitor during the entire training session (V800; Polar Unk, Oy, Kempele, Finland). The researchers supervised each training session. The researchers monitored heart rates during all exercise intervention sessions. The wearable heart rate monitors used in the study were set to sound an alarm if the heart rate fell below or exceeded the target HRmax range, allowing the researchers to ensure the intensity of exercise during all sessions. In addition, the wearable devices of participants were programmed to activate an alarm to maintain heart rates within the target HRmax range, and participants were pre-educated on the significance of these alarms.

### 2.3 Anthropometric assessments

Each participant’s weight was measured automatically while comfortably standing with feet slightly apart on the instrument and wearing simple clothing. Barefoot stature was measured in centimeters without shoes, heels together, and with the back of the subject parallel to the stadiometer. Even with several limitations in accuracy, bioelectrical impedance analysis (BIA) still has high sensitivity and specificity in estimating body composition in children (Kyle et al., 2015). Body composition was determined using Inbody J10 (Biospace Corp., Seoul, Korea), evaluating body weight (kg), body fat percentage (BFP, %), and muscle mass (kg). The formula for BMI used was weight in kilograms divided by height in meters squared.

### 2.4 Physical fitness tests

Physical fitness tests were conducted before and after the exercise period. Back-muscle strength was measured using a back muscle dynamometer (TKK-5002, Takei Corp., Japan). The participants stood on the platform, bent their upper body forward approximately 30°, and then exerted maximum effort to straighten their upper body while pulling the handle. Grip strength test (kilograms; kg) measures muscular strength using the grip dynamometer (TKK-5403, Takei Corp., Niigata, Japan). During the test, the children stand in a comfortable position with arms straightened on either side of the torso at 15° while applying force, and the relative grip strength is recorded in units per 0.1 kg. Sit-up test (repetitions) measures muscular endurance by counting the number of times the upper body is moved up to touch the knees with the elbows (knees bent at a 90° angle) within 60 s. Sit-and-reach test (centimeters; cm) measures lower body flexibility by having the participant sit with knees straight and attempt to reach forward with both hands extended as far as possible toward the sit-and-reach meter (TKK-5404, Takei Corp., Japan). A 20-m shuttle run test (repetitions) for measuring cardiorespiratory fitness (aerobic capacity) involves continuous running back and forth between two parallel lines set 20-m apart in time audio signals. The test measures the number of stages completed, with the pace increasing every minute. Closed-eye foot balance test (seconds; s) measures balancing ability by recording the time a participant can stand comfortably on one leg with hands on the waist and eyes closed.

### 2.5 Blood sampling and analysis

To investigate the potential effects of the exercise intervention, blood samples were obtained from all participants at baseline and after 16 weeks. Participants were instructed to fast for a minimum of 8 h prior to each sample collection. Between 8 a.m. and 10 a.m., a clinical pathologist collected 10 mL of blood from the antecubital vein. The blood samples were then centrifuged at 3,500 g at 4°C and 3,000 rpm for 10 min using a Sorvall Micro 17 centrifuge (Thermo Fisher Scientific, MA, United States). After centrifugation, the serum was separated and stored at −80°C until the analysis. Serum concentrations of C-peptide (ng/mL), resistin (ng/mL), IGF-1 (ng/mL), and GH (ng/mL) were quantified following the manufacturer’s recommendations (Abcam, Cambridge, MA, United States).

### 2.6 Statistical analysis

The optimal total sample size, was calculated using statistical software G*Power 3.1, considering a small effect size of F = 0.25 (default), a power of 0.80, and an alpha of 0.05. All statistical tests were conducted with an alpha level of *p* < 0.05. First, we conducted a normality test on our research results and confirmed that they followed a normal distribution. A two-way analysis of variance with repeated measures was used to compare changes pre- and post-exercise within and between groups, followed by a *post hoc* Bonferroni’s test. The size of the effect between pre- and post-test data was calculated using Cohen’s d (|0.20| ≤ small < |0.50| < medium < |0.80| ≤ large) (Cohen, 2013). Correlations between changes in body composition, physical fitness level, blood biomarkers, and C-peptide were determined using Pearson or Spearman correlation coefficients. Associations of blood biomarkers with body composition and physical fitness factors were analyzed using stepwise multiple linear regression models. Model 1 was adjusted for the non-obese group, and Model 2 was adjusted for the obese group. The inclusion and exclusion criteria for these analyses were based on the significance level of the F-value, set to 0.05. The best equation was selected based on the highest multiple correlation coefficient (R2).

## 3 Results

### 3.1 Regulation of body composition balance through exercise

Body composition before and after the EP is shown in Table 1. Before the EP, body weight (*F* = 26.132, *p* = 0.000), BMI scores (*F* = 50.291, *p* = 0.000), and BFP (*F* = 12.800, *p* = 0.001) were significantly higher in the obese group by 29.3%, 22.8%, and 24.0%, respectively, than those in the non-obese group. After the EP, the obese group showed significant reduction in BFP (*F* = 23.200, *p* = 0.000) and a significant increase in muscle mass (*F* = 8.513, *p* = 0.007). The difference in BFP showed a substantial reduction between the two groups, decreasing from a pre-EP BFP of 24.0% to a post-EP BFP of 0.28%. Another notable change was observed in muscle mass, transitioning from a pre-EP difference of 3.95% to a post-EP difference of 28.8%.

**TABLE 1:** Body composition of two groups before and after the 16-week exercise program.

Variables	Groups	Pre-EP	Post-EP	Effect size Cohen’s *d*	Interaction	Main effect	
		(Mean ± SD)	(Mean ± SD)				
Height (cm)	Non-obesity	144.55 ± 9.94	147.78 ± 9.05^***^	0.36 (*small*)	*F = 3.445, p* = 0.073	Time *F* = 139.157 ^†††^ *p* = 0.000	
	Obesity	148.44 ± 7.53	152.88 ± 7.33^***^	0.59 (*medium*)			
	*Difference*	2.69% ± 8.63%	3.45% ± 7.88%	0.09 (*no effect*)			
Weight (kg)	Non-obesity	45.31 ± 7.02	46.94 ± 7.09	0.23 (*small*)	*F = 0.519* *p* = 0.477	Group *F* = 26.132 ^†††^ *p =* 0.000	
	Obesity	58.57 ± 6.93	59.31 ± 8.55	0.11 (*no effect*)			
	*Difference*	29.3% ± 22.2%	26.3% ± 24.0%	−0.14 (*no effect*)			
BMI (kg/m^2^)	Non-obesity	21.62 ± 1.91	21.39 ± 1.67	−0.12 (*no effect*)	*F = 1.538* *p* = 0.224	Group *F* = 50.291 ^†††^ *p* = 0.000	
	Obesity	26.54 ± 1.61	25.56 ± 2.76	−0.61 (*medium*)			
	*Difference*	22.8% ± 11.7%	19.5% ± 15.2%	−0.28 (*small*)			
BFP (%)	Non-obesity	30.33 ± 6.23	29.08 ± 7.38	−0.20 (*small*)	*F* = 12.800 ^†††^ *p* = 0.001	Time *F* = 23.200 ^†††^ *p* = 0.000	
	Obesity	37.61 ± 8.01	29.16 ± 5.77^***^	−1.06 (*large*)			
	*Difference*	24.0% ± 33.8%	0.28% ± 32.2%	−0.70 (*large*)			
Muscle mass (kg)	Non-obesity	21.28 ± 9.57	24.02 ± 10.02	0.29 (*small*)	*F = 2.355* *p*= 0.135	Time *F*= 8.513 ^††^ *p*= 0.007	
	Obesity	22.12 ± 9.18	30.94 ± 12.15^*^	0.96 (*large*)			
	*Difference*	3.95% ± 62.3%	28.8% ± 66.7%	0.40 (*small*)			

*Summary of repeated measures two-way repeated measures ANOVA, and Cohen’s d effect size analysis for body composition data*.

*p< 0.05.,***p< 0.001 vs. Pre-EP, ^††^ *p*< 0.01, ^†††^ *p*< 0.001.

*Note*: BMI, body mass index; BFP, body fat percentage; EP, exercise program; SD, standard deviation.

*“Difference”* represents a difference between the two groups in the column for each variable.

Cohen’s d effect size range: *no effect*for *d*< |.20|; *small*for |.20| ≦ *d*< |.50|, *medium*for |.50| ≦ *d*< |.80|, *large*for |.80| ≦ *d*.

### 3.2 Physical fitness capacity improvement through exercise

Physical fitness before and after the EP is shown in Table 2. No significant interactions between the time and group were observed for any physical fitness parameter. However, a significant group effect was detected in back-muscle strength (*F* = 5.806, *p* = 0.022), handgrip strength (*F* = 7.159, *p* = 0.012), and sit-ups (*F* = 4.441, *p* = 0.043), indicating differences in strength capacity between the two groups. Following the EP, a significant main effect of time was detected for all parameters, including back-muscle strength (*F* = 14.999, *p* = 0.001), handgrip strength (*F* = 38.083, *p* = 0.000), sit-ups (*F* = 65.683, *p* = 0.000), flexibility (*F* = 29.130, *p* = 0.000), and cardiovascular fitness (*F* = 50.372, *p* = 0.000), indicating an overall increase in physical fitness levels during the EP in both groups. The range of effect sizes for all physical fitness parameters in the obese group (*d* = 0.70–2.83) exceeded those in the non-obese group (*d* = 0.20–1.61). Particularly noteworthy was the balance level in the obese group (*d* = 0.89), which was 4.45-fold higher than that in the non-obese group (*d* = 0.20).

**TABLE 2:** Physical fitness levels of pre- and post-exercise program.

Variables	Group	Pre-EP	Post-EP	Effect size Cohen's *d*	Interaction	Main effect
		(mean ± SD)	(mean ± SD)			
Back-muscle strength (kg)	Non-obesity	32.34 ± 12.74	36.82 ± 17.08^*^	0.35 (*small*)	*F=1.446,* *p*=0.238	Time *F*=14.999, ^†††^ *p*=0.001
	Obesity	42.22 ± 12.21	50.75 ± 17.16^**^	0.70 (*medium*)		Group *F*=5.806 ^†^ *p*=0.022
	*Difference*	30.56 ± 55.89 %	37.83 ± 68.06 %	0.13 (*no effect*)		
Handgrip strength (kg)	Non-obesity	17.13 ± 5.17	19.59 ± 4.48^**^	0.48 (*small*)	*F=1.447, p*=0.238	Time *F*=38.083, ^†††^ *p*=0.000
	Obesity	20.35 ± 3.68	24.01 ± 3.77^***^	1.00 (*large*)		Group *F*=7.159, ^†^ *p*=0.012
	*Difference*	18.80 ± 37.48 %	22.56 ± 30.33 %	0.10 (*no effect*)		
Sit-up (times)	Non-obesity	20.35 ± 6.02	30.06 ± 10.90^***^	1.61 (*large*)	*F=0.008, p*=0.224	Time *F*=65.683, ^†††^ *p*=0.000
	Obesity	10.94 ± 6.08	24.69 ± 8.36^***^	2.26 (*large*)		Group *F*=4.441 ^†^ *p*=0.043
	*Difference*	46.24 ± 44.21 %	17.86 ± 46.15 %	−0.64 (*large*)		
Flexibility (cm)	Non-obesity	10.18 ± 6.54	13.11 ± 8.57^**^	0.45 (*small*)	*F=10.166, p*=0.263	Time *F*=29.130, ^†††^ *p*=0.000
	Obesity	5.39 ± 5.20	9.89 ± 4.66^***^	0.87 (*large*)		
	*Difference*	47.05 ± 87.47 %	24.56 ± 76.12 %	−0.26 (*small*)		
Cardiovascular fitness (times)	Non-obesity	20.76 ± 11.95	34.29 ± 23.12^***^	1.13 (*large*)	*F=2.576, p*=0.119	Time *F*=50.372, ^†††^ *p=*0.000
	Obesity	16.06 ± 7.58	37.50 ± 18.60^***^	2.83 (*large*)		
	*Difference*	22.64 ± 69.40 %	9.36 ± 86.77 %	−0.19 (*no effect*)		
Balance (sec)	Non-obesity	47.25 ± 52.81	57.62 ± 36.25	0.20 (*small*)	*F=2.765,* *p*=0.106	Time *F*=17.544, ^†††^ *p*=0.000
	Obesity	27.50 ± 26.92	51.52 ± 23.29^***^	0.89 (*large*)		
	*Difference*	41.80 ± 133.87 %	89.41 ± 69.27 %	0.36 (*small*)		

*Summary of repeated measures two-way ANOVA, and Cohen’s d effect size analysis for physical fitness data*.

*p < 0.05, ***p < 0.001 vs. Pre-EP, ^††^ *p* < 0.01, ^†††^ *p* < 0.001.

*Note*: EP, exercise program; SD, standard deviation.

*“Difference”* represents a difference between the two groups in the column for each variable.

Cohen’s d effect size range: *no effect* for *d* < |.20|; *small* for |.20| ≦ *d* < |.50|, *medium* for |.50| ≦ *d* < |.80|, *large* for |.80| ≦ *d*.

### 3.3 Improvement of C-Peptide, resistin, IGF-1, and GH levels in childhood obesity

Circulating hormone factors before and after the EP are shown in Table 3. Before the EP, individuals with obesity exhibited significantly higher C-peptide levels that those without obesity (Table 3). Following the EP, C-peptide levels significantly decreased in both groups (*F* = 48.101, *p* = 0.000). While no significant main effect of group in resistin was observed, the EP significantly reduced resistin levels in the obese group (*F* = 27.291, *p* = 0.000), but not in the non-obese group. Baseline IGF-1 levels were comparable between both groups and significantly increased after undergoing the EP (*F* = 42.333, *p* = 0.000). However, different responses were noted in GH levels between the non-obese and obese groups before and after undergoing the EP. GH levels in the obese group were significantly lower than those in the non-obese group (*F* = 5.739, *p* = 0.023), and the EP led to increased GH levels in both groups (*F* = 9.891, *p* = 0.004).

**TABLE 3:** Change of blood biomarkers after 16 weeks of the exercise training.

Variables	Group	Pre-test	Post-test	Effect size Cohen's *d*	Interaction	Main effect
		Mean ± SD	Mean ± SD			
C-peptide (ng/ml)	Non-obesity	2.36 ± 0.91	1.77 ± 0.76^*^	-0.65	*F*=10.294, ^†^ *p*=0.003	Time *F*=48.101, ^†††^ *p*=0.000 Group *F*=15.333, ^†††^ *p=*0.000
	Obesity	4.58 ± 1.75	2.96 ± 1.67^***^	−0.93		
Resistin (ng/ml)	Non-obesity	11.18 ± 8.07	10.76 ± 7.19	−0.05	*F*=15.481, ^†††^ *p*=0.000	Time *F*=27.291, ^†††^ *p=*0.000
	Obesity	14.05 ± 11.24	11.06 ± 9.69^***^	−0.27		
IGF-1 (ng/ml)	Non-obesity	265.56 ± 128.11	311.81 ± 150.53^***^	0.36	*F*=0.017, *p*=0.897	Time *F*=42.333, ^†††^ *p*=0.000
	Obesity	224.74 ± 114.70	272.89 ± 138.26^***^	0.42		
GH (ng/ml)	Non-obesity	3.91 ± 3.09	4.80 ± 3.17^*^	0.29	*F*=0.068, *p*=0.796	Time *F*=9.891, ^††^ *p*=0.004 Group *F*=5.739, ^†^ *p=*0.023
	Obesity	1.76 ± 2.25	2.51 ± 2.34^*^	0.33		

*Summary of repeated measures two-way repeated measures ANOVA and Cohen’s d effect size analysis for blood biomarkers*.

*p < 0.05, ***p < 0.001 vs. Pre-EP, ^†^ *p* < 0.05, ^††^ *p* < 0.01, ^†††^ *p* < 0.001.

*Note*: *IGF-1, Insulin Growth factor–1, GH, growth hormone;* EP, exercise program; SD, standard deviation.

*“Difference”* represents a difference between the two groups in the column for each variable.

Cohen’s d effect size range: *no effect* for *d* < |.20|; *small* for |.20| ≦ *d* < |.50|, *medium* for |.50| ≦ *d* < |.80|, *large* for |.80| ≦ *d*.

### 3.4 C-peptide and IGF-1 association with changes in body composition and physical fitness following exercise

**FIGURE 1:**
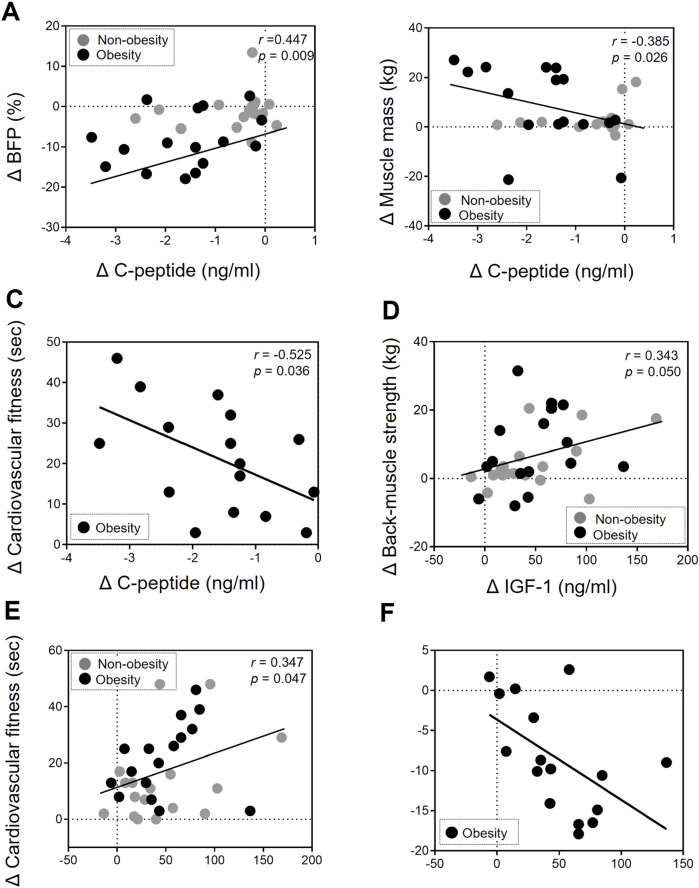
Association of changes in body composition and physical fitness with C-peptide and IGF-1 levels after exercise intervention. (**A, B**) The change in (Δ) C-peptide levels was correlated with the change (Δ) in BFP (*r* = 0.447, *p* = 0.009) and muscle mass (*r* = −0.385, *p* = 0.026) after undergoing the EP in the non-obesity and obesity group. **(C)** The change in (Δ) C-peptide levels was correlated with the change (Δ) in cardiovascular fitness (*r* = −0.525, *p* = 0.036) after undergoing the EP in the obesity group. (**D, E**) The change (Δ) in IGF-1 levels was correlated with the change (Δ) in back-muscle strength (*r* = 0.343, *p* = 0.05) and cardiovascular fitness levels (*r* = 0.347, *p* = 0.047) after undergoing the EP in the non-obesity and obesity group. **(F)** The change in (Δ) IGF-1 levels was correlated with the change (Δ) in BFP (*r* = −0.542, *p* = 0.003) after undergoing the EP in the obesity group. A correlation analysis was performed (Pearson’s correlation coefficients). Each gray and back circle represents a participant in the non-obesity (n = 17) and obesity (n = 16) groups, respectively.

To elucidate the impact of biochemical markers in the EP-improved body composition and physical fitness level, we conducted statistical correlations using delta values (difference) of all parameters pre- and post-EP. In both groups, changes in C-peptide levels were significantly associated with improvements in BFP (*r* = 0.447, *p* = 0.009; Figure 1A) and muscle mass (*r* = −0.385, *p* = 0.026; Figure 1B). A decrease in C-peptide levels in the obese group was associated with a change in cardiovascular fitness (*r* = −0.525, *p* = 0.036; Figure 1C). Changes in IGF-1 levels were significantly associated with EP-induced improvements in physical fitness, including back-muscle strength (*r* = 0.343, *p* = 0.050; Figure 1D) and cardiovascular fitness level (*r* = 0.347, *p* = 0.047; Figure 1E) in both groups. Regarding the association with body composition, a decrease in BFP was associated with a change in IGF-1 levels in the obese group (*r* = −0.542, *p* = 0.003; Figure 1F).

### 3.5 Factors affecting changes in obesity-related biochemical markers in the non-obesity and obesity groups

To analyze the factors associated with biochemical markers after undergoing the EP, we conducted four iterations of multiple linear regression analyses using the delta values in each group (Table 4). In the non-obese group, no factors affecting C-peptide and resistin levels were detected; however, back-muscle strength (β = 3.045, *p* < 0.05) influenced IGF-1, and height (β = 0.45, *p* < 0.05) affected GH levels. In the obese group, a significant association was observed between C-peptide levels and cardiovascular fitness level (β = −0.054, *p* < 0.001), as well as glucose levels (β = 0.083, *p* < 0.001). In addition, IGF-1 (β = −2.936, *p* < 0.05) had a reducing effect on BFP. These findings suggest that C-peptide and IGF-1 may contribute to improving body composition and physical fitness levels through regular exercise in adolescent with obesity.

**TABLE 4:** Factors affecting obesity-related biochemical markers by multiple regression analysis (stepwise) in each group.

Model 1	C-peptide			Resistin				IGF-1				GH		
	Beta	SE	VIF^†^	Beta	SE	VIF^†^		Beta	SE	VIF^†^		Beta	SE	VIF^†^
	None			None			Back-muscle strength	3.045*	1.336	1.000	Height	0.453^*^	0.192	1.000
Adjusted R^2^								0.208				0.221		

Model 1	C-peptide			Resistin				IGF-1				GH		
	Beta	SE	VIF^†^	Beta	SE	VIF^†^		Beta	SE	VIF^†^		Beta	SE	VIF^†^
Cardio-vascular Fitness	−0.054^***^	0.009	1.048	None			BFP	−2.936^*^	1.216	1.000	None		
Adjusted R^2^	0.806								0.243					

^†^Variance Inflation Factor: VIF, When VIF would be more than 10, the impact of multicollinearity could be strong.

*Note:*IGF-1: *insulin*Growth Factor-1, GH: Growth Hormone, BFP = Body fat percentage.

Model 1: Non-obesity group. Model 2: Obesity group. *p< 0.05, ***p< 0.001.

## 4 Discussion

In this study, we explored the impact of a supervised exercise intervention on body composition and physical fitness in adolescent boys with obesity and the potential reduction in obesity-related circulating hormones. In addition, we examined the associations between biomarkers and exercise-induced variables. Our results demonstrated positive effects of the EP on body composition and physical fitness parameters. Moreover, this study is novel in demonstrating the promising roles of blood levels of C-peptide and IGF-1 in contributing to exercise-induced improvements in body composition and physical fitness in early adolescent boys with obesity. This study specifically links biochemical changes to enhancements in muscle strength and cardiovascular fitness through a structured 16-week EP.

Body composition analysis is a cornerstone for identifying childhood obesity and assessing the clinical efficacy of interventions. Consistent with previous studies, our findings demonstrated that the EP intervention effectively reduced BMI scores and BFP in children with obesity. Conversely, no significant changes in body composition were observed among children without obesity (Tan et al., 2016; 2017). A recent randomized controlled trial compared the effect of aerobic exercise intensity on adolescent boys with obesity and found that reductions in BFP were more significant following continuous moderate-intensity training than high-intensity interval training (Meng et al., 2022). The EP training in our study may have similar effects on body fat reduction with moderate-intensity aerobic exercise. A notable finding was the increase in muscle mass among children with obesity participating in the EP, particularly those engaging in resistance training. This aligns with the established efficacy of a 16-week resistance exercise in enhancing lean body mass by approximately 7.4% in the context of obesity treatment in adolescent boys with obesity (Shaibi et al., 2006). However, in our study, obese children who participated in the EP increased their strength by approximately 28% during the EP. Although the exact mechanisms behind this strength gain cannot be determined, the higher frequency of exercise (3 days per week) may have contributed to the substantial differences in changes in lean body mass. This supports the notion that a higher volume of exercise intervention may have improved body composition, particularly muscle gain.

Previous research has established low childhood physical fitness as a critical determinant of health, with lower fitness levels linked to increased risks of metabolic and cardiovascular diseases (Ortega et al., 2008). Children with obesity and elevated high BMI scores often exhibit reduced physical capacity, including diminished endurance and muscular fitness (Musálek et al., 2020). Our findings corroborate this, revealing significantly lower muscle strength in children with obesity than in children without obesity. Aligned with a systematic review demonstrating improved muscular fitness following physical activity intervention in children with obesity (Thivel et al., 2016), our 16-week EP significantly enhances overall physical fitness, encompassing cardiovascular endurance, muscular strength, muscular endurance, flexibility, and balance. These findings suggest that the EP is an effective intervention for improving health outcomes in adolescent boys with obesity by targeting health-related physical fitness components through a balanced combination of aerobic, resistance, and sports activities.

Obesity-induced insulin resistance in children is considered to be an important pathophysiologic indicator linking obesity and metabolic derangement (Lee, 2006). In the context of monitoring insulin resistance and sensitivity, the measurement of C-peptide levels in the blood also serves as a significant biomarker of diabetes since it is secreted in equimolar amounts with insulin (Maddaloni et al., 2022). Previous studies consistently indicate that higher insulin production correlates with increased C-peptide levels (Steiner et al., 2009; Jones and Hattersley, 2013). Our study found that the obese group exhibited higher C-peptide levels than the non-obese group, which is consistent with insulin-based indices. A recent systemic review and meta-analysis published in 2023 showed the positive effects of exercise interventions on insulin resistance in children and adolescents with overweight and obesity (Kazeminasab et al., 2023). Similar to the effects of exercise on insulin resistance, our study also observed that the EP intervention reduced C-peptide levels in obese children, with these declines correlating with changes in BFP and muscle mass. This study supports the notion that the positive effects of exercise on body composition and skeletal muscle metabolism beneficially improved insulin sensitivity and decreased insulin resistance in obese children, which subsequently resulted in lower insulin secretion.

Our study showed that changes in C-peptide levels were associated with improved physical fitness following the EP intervention. The relationship between C-peptide levels and physical fitness parameters requires further exploration. A recent meta-analysis and systematic review suggested that serum C-peptide is highly associated with an increased risk of cardiovascular disease (CVD) (Ahmadirad et al., 2023). Higher physical fitness, particularly cardiovascular fitness, has been associated with a favorable risk profile for CVD in children and adolescents (Ruiz et al., 2016). In our study, the reduction in C-peptide along with increased physical fitness may contribute to a reduction in obesity-related CVD risk.

Our study had several limitations. First, the relatively small sample size restricts the generalizability of our findings to larger populations of children with obesity. Future studies with larger sample sizes are essential to validate our results and provide more robust evidence. Second, the EP was limited to 16 weeks. Long-term follow-up studies are necessary to assess the sustainability and lasting effects of exercise on body composition and physical fitness in children with obesity. Third, our study focused on the effects of exercise on C-peptide and IGF-1 levels as mediators of the observed outcomes; however, other factors and mechanisms may contribute to the regulation of body composition and physical fitness in children with obesity. Further studies are required to identify additional biomarkers and pathways involved in these processes. Fourth, our study lacked sex-specific analysis, as we did not separately analyze data for girls. Future research should explore sex-specific effects of the EP to better understand its impact on body composition, physical fitness, and circulating hormones. Fifth, our study did not evaluate pubertal status of the participating boys. The causal relationship between pubertal timing and outcome measurements remains uncertain. Sixth, to estimate body composition, our study used the BIA method instead of a gold-standard measure like dual-energy X-ray absorptiometry. Finally, our study did not examine the potential effects of dietary factors or lifestyle changes on the outcomes. The effects of exercise on body composition and physical fitness may interact with other variables such as diet quality and adherence to EPs. Future studies should consider these factors when exploring the combined effects. Despite these limitations, our study provides valuable insights into the role of C-peptide and IGF-1 in the improvement of body composition and physical fitness through exercise intervention in early adolescent boys with obesity.

## 5 Conclusion

In conclusion, our study revealed that exercise is crucial in regulating body composition and physical fitness in early adolescent boy with obesity. As circulating biomarkers of obesity, C-peptide and IGF-1 were associated with the effects of exercise on these outcomes. Our findings support the development of targeted exercise interventions and reinforce the importance of exercise as an integral component of child and adolescent obesity management programs.

## Data Availability

The raw data supporting the conclusions of this article will be made available by the authors, without undue reservation.
